# COVID-19 Vaccine Acceptance Behavior among Hispanics/Latinxs in Nevada: A Theory-Based Analysis

**DOI:** 10.3390/healthcare11050688

**Published:** 2023-02-26

**Authors:** Tara Marie Nerida, Manoj Sharma, Brian Labus, Erika Marquez, Chia-Liang Dai

**Affiliations:** 1 Department of Social and Behavioral Health, University of Nevada, Las Vegas, NV 89119, USA; 2 Department of Internal Medicine, Kirk Kerkorian School of Medicine, University of Nevada, Las Vegas, NV 89102, USA; 3 Department of Epidemiology and Biostatistics, University of Nevada, Las Vegas, NV 89119, USA; 4 Department of Environmental and Occupational Health, University of Nevada, Las Vegas, NV 89119, USA; 5 Department of Teaching and Learning, University of Nevada, Las Vegas, NV 89119, USA

**Keywords:** COVID-19, vaccine acceptance, initiation, sustenance, multi-theory model of health behavior change, Hispanic/Latinx

## Abstract

Hesitancy toward the COVID-19 vaccine has hindered its rapid uptake among the Hispanic and Latinx populations. The study aimed to use the Multi-Theory Model (MTM) for health behavior change to explain the intention of initiating and sustaining the behavior of COVID-19 vaccination among the Hispanic and Latinx populations that expressed and did not express hesitancy towards the vaccine in Nevada. Using a quantitative cross-sectional and survey-based research study design, data were collected using a 50-item questionnaire and analyzed using multiple linear regression modeling. Of 231 respondents, participatory dialogue (b = 0.113, *p* < 0.001; b = 0.072, *p* < 0.001) and behavioral confidence (b = 0.358, *p* < 0.001; b = 0.206, *p* < 0.001) displayed significant associations with the initiation of COVID-19 vaccine acceptance among vaccine-hesitant and non-vaccine-hesitant individuals. Emotional transformation (b = 0.087, *p* < 0.001; b = 0.177, *p* < 0.001) displayed a significant association with the sustenance of COVID-19 vaccine acceptance among vaccine-hesitant and non-vaccine-hesitant individuals. Results from this study provide evidence that the MTM is a useful tool in predicting COVID-19 vaccine acceptance behavior among Hispanics and Latinxs in Nevada, and it should be used in intervention designs and messaging to promote vaccine uptake.

## 1. Introduction

In 2020, the COVID-19 pandemic quickly became a global public health issue that has drastically impacted many lives, including individuals’ and communities’ health, economic shifts, and social and physical restrictions. The repercussions that the COVID-19 virus inflicted on the world have left many populations trying to get back to a “normal” life to this day.

The drastic impact COVID-19 had on the United States left over ninety million cases and over one million deaths as of 4 August 2022 [1]. Additionally, the COVID-19 pandemic exposed the disproportionate health impacts on vulnerable populations, including the inequalities affected by income, age, race, sex, and geographic location [2,3]. This was particularly evident among the Hispanic and Latinx populations across the United States. Compared to White non-Hispanic people, Hispanic or Latinx people are 1.5 times more likely to be diagnosed with COVID-19, 2.3 times more likely to be hospitalized because of COVID-19, and 1.1 times more likely to die from COVID-19 [4]. Due to having the highest uninsured rates, the majority of these populations being unauthorized or undocumented immigrants and ineligible for Medicaid or other government benefits, and having significant language barriers, the Hispanic and Latinx populations have faced many challenges that make them vulnerable to COVID-19 and the drastic effects that have impacted them [5].

As of June 2021, three COVID-19 vaccines had been approved for Emergency Use Authorization (EUA) by the U.S. Food and Drug Administration (FDA): Pfizer-BioNTech (BNT161b2), Moderna (mRNA-1273), and Janssen (Ad26.COV2.S) [6]. According to studies, the Pfizer, Moderna, and Janssen vaccines showed 95%, 94.1%, and 66% efficacy, respectively, at preventing illness in clinical lab settings, including severe disease leading to hospitalization and death [7,8,9]. The Food and Drug Administration [FDA] fully approved the Pfizer-BioNTech COVID-19 vaccine on 23 August 2021 (FDA, 2021) and the Moderna COVID-19 vaccine on 31 January 2022 [10]. However, hesitancy toward the COVID-19 vaccine has hindered its rapid uptake.

Vaccine hesitancy is defined as a “delay in acceptance or refusal of vaccines despite availability of vaccine services” [11] and has emerged as a public health issue threatening the end of the COVID-19 pandemic. Many are hesitant to receive the COVID-19 vaccine for various reasons, including the fear of vaccine side effects, the safety of the vaccine, and its effectiveness given how new the vaccine was [12]. Another threat to vaccine acceptance is the “infodemic”, which the World Health Organization defines as “an overabundance of information and the rapid spread of misleading or fabricated news, images, and videos” [13]. This infodemic has amplified the amount of misinformation being spread about the COVID-19 vaccine, which results in increased hesitancy among vulnerable populations who have utilized social media as a major form of receiving information about the vaccine [14,15,16,17]. Addressing these hesitancies and building vaccine confidence is key to increasing vaccine uptake.

When the first COVID-19 vaccines were introduced and just about to be released in the United States, the December 2020 Kaiser Family Foundation COVID-19 Vaccine Monitor found that among Hispanic adults, 61% trusted the safety and effectiveness of the vaccine, 61% believed that the vaccine would be distributed equally, and 60% were confident that the development of the vaccines had considered the needs of the Hispanic and Latinx people [18]. This finding showed that the Hispanic and Latinx populations may have been interested in receiving the vaccine early on. However, after the release of the plans for vaccine distribution across the United States, because many of the Hispanic and Latinx populations were not eligible for the vaccine right away, the long wait to get vaccinated may have allowed for more time to increase vaccine hesitancy. This may have also been influenced by several factors, such as misinformation, myths, citizenship status, language barriers, work schedules, lack of understanding of virtual technologies to schedule vaccine appointments, etc., which are responsible for this disparity [19]. Although many members of the Hispanic and Latinx population are accepting of getting the COVID-19 vaccine, others are still hesitant due to historical and pre-existing experiences that have previously affected the hesitancy of getting vaccinated, including lower access to adequate healthcare providers for minority populations, historical mistrust, cost-related concerns, and lower awareness and education about the importance of the vaccine [20]. While not a comprehensive list, these are some of the factors that have affected the uptake of routine immunizations that have been available for years.

The acceptance of the COVID-19 vaccines has also been linked with historical reluctance to accept other routine immunizations, especially seasonal influenza (flu) vaccines [21,22]. The Hispanic and Black communities in the United States have traditionally had lower rates of flu vaccine coverage compared to Whites [21]. Survey results of one study show that those who did not intend to get the COVID-19 vaccine when it became available had 79% lower odds (aOR = 0.21) of receiving a flu vaccine in the previous year [22].

The intent to get vaccinated is ultimately determined by values, cultures, and experiences, which include how Hispanics and Latinxs rely heavily on trusted voices within the communities to provide their expertise about vaccinations [23]. Many cultures have various views on vaccination, including the COVID-19 vaccine, which has affected vaccine uptake [23]. Similarly, trust and mistrust in influential individuals within specific cultures has been shown to affect the uptake of the COVID-19 vaccine. Lower levels of trust toward science and state-sponsored health programs among ethnic minorities, including the Hispanic and Latinx populations, African Americans, Native Americans, Native Hawaiians, and Alaskan Natives, are a direct result of previous or negative experiences with unethical healthcare research and an unethical healthcare system, as well as an under-representation of ethnic minorities in research [24]. This may include colonization, eugenics, and medical experiments that inhibit the trust of Hispanics and Latinxs, among other minorities, towards the healthcare system [24,25,26]. In addition, Hispanic and Latinx people have also demonstrated vaccine hesitancy influenced by cultural factors, such as moral concerns related to the belief that vaccine manufacturers used abortion-derived fetal cell lines or the belief that religious prayers should be preferred over the use of medicine [27,28]. Because the Hispanic and Latinx populations around the United States are significantly vulnerable to COVID-19 complications, hospitalizations, and deaths [4], further investigation is needed to understand their perceptions and intentions of receiving the COVID-19 vaccine and completing the vaccine series compared to other racial and ethnic groups. Based on this information, four problems were identified to be addressed by public health professionals: (1) there are high rates of COVID-19 in Hispanics and Latinxs in the United States and Nevada; (2) there are low rates of vaccination in Hispanics and Latinxs in the United States and Nevada; (3) there is little literature, especially theory-based literature, focusing on the determinants of COVID-19 vaccination in Hispanics and Latinxs; and (4) there is a problem of Hispanics and Latinxs not being interested in or following through with taking the second dose or booster vaccines.

The purpose of this study was to use a fourth-generation theory-based approach of the Multi-Theory Model (MTM) of health behavior change to explain the intention of initiating COVID-19 vaccination among the Hispanic and Latinx populations that expressed and did not express hesitancy toward the vaccine in Nevada. The covariates that were controlled for because of their possible effects on COVID-19 vaccination uptake status were age, race, gender, education level, religion, income, and employment status [29].

## 2. Materials and Methods

### 2.1. Population

The study was conducted out of the University of Nevada, Las Vegas between May and August 2022. The population being sampled was Hispanic and Latinx individuals residing in Nevada from the years 2021 to 2022. In order to determine the required sample size for the multiple regression, an a priori sample size was calculated using the G*Power, Version 3.1.9.6 for Mac [30,31]. The parameters set for this calculator for regression were an alpha level of 0.05, power at 0.80, an estimated effect size of 0.15 (medium), and three predictors (for the three constructs in each of the initiation and sustenance components of the MTM). This yielded a required sample size of 77. To account for any covariates that may be found as significant, the sample size was inflated by approximately 20%, which is around 92 for each of the hesitant and non-hesitant groups. Thus, the total sample size proposed was at least 184, which was also considered sufficient for confirmatory factor analysis [32].

Inclusion criteria for participation in the study were: (1) of Hispanic or Latinx descent; (2) age 18 years or older; (3) currently residing in Nevada; and (4) providing informed consent to participate if the study was exempt. Participants who did not meet the above inclusion criteria and those who were mandated to receive the COVID-19 vaccine for employment or school requirements were excluded from the study.

### 2.2. Theoretical Framework

**Figure 1:**
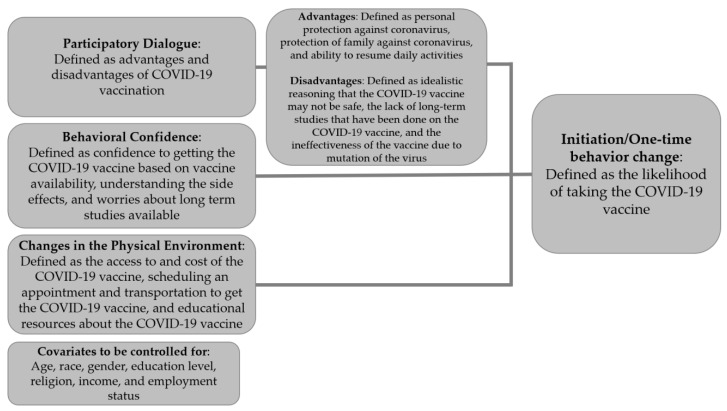
Constructs in the initiation of health behavior change in the Multi-Theory Model of health behavior change.

**Figure 2:**
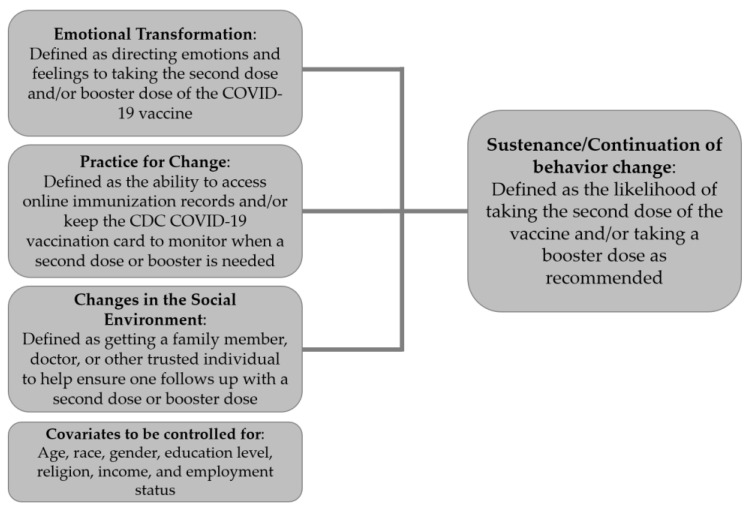
Constructs in the sustenance of health behavior change in the Multi-Theory Model of health behavior change.

The present study uses the Multi-Theory Model (MTM) of health behavior change as the theoretical framework to explore vaccine acceptance behaviors among Hispanics and Latinxs in Nevada due to its unique ability to explain the intention and sustenance of behavior change [33]. There are two components of the MTM that facilitate health behavior change: (1) initiation of the behavior change, and (2) sustenance or continuation of the health behavior change [33]. Initiation of the behavior change refers to a one-time or short-term change that progresses a person from one behavior to another [33]. Sustenance or continuation of the health behavior change is the long-term change that continues after initiation is enacted [33]. The constructs of participatory dialogue (i.e., the advantages and disadvantages of health behavior change and the dialogue facilitated by a health educator to create change), behavioral confidence (i.e., the culturally-specific term that refers to the confidence or belief that the person is capable of initiating and achieving the desired behavior change), and changes in the physical environment (i.e., the physical surroundings that provide resources for the person to initiate the behavior change) will contribute to the initiation of intended behavior [33]. Figure 1 shows how the constructs of initiation interact and were operationalized in this study. The constructs of emotional transformation (i.e., when a person transforms or converts their emotions towards the health behavior change they are trying to sustain), practice for change (i.e., when the person continuously evaluates and adjusts the strategies, overcomes the barriers, and remains focused on maintaining that behavior change), and changes in the social environment (i.e., the social support, either natural or artificial, from the environment that creates a positive relationship with sustained behavior change) will lead to the sustenance of the intended behavior [33]. Figure 2 shows how the constructs of sustenance interact and were operationalized in this study.

### 2.3. Instrumentation

The survey instrument consisted of 50 total items and was developed based on the MTM theoretical framework to assess vaccine acceptance behavior. One item assessed the current state of vaccine hesitancy (i.e., do you currently have any hesitancy in taking the COVID-19 vaccine?), and two items assessed if the person had already completed at least one dose or the full series of the COVID-19 vaccine dosage. Fourteen items assessed socioeconomic characteristics (i.e., age, zip code of residence, gender, ethnicity and Hispanic/Latinx subgroup, education level, etc.), two of which were optional questions at the end of the survey, as they asked about political affiliation and citizenship status. Religion is an important aspect of the lives of the Hispanic/Latinx population; therefore, it was important that the item addressing religion included the most common religious affiliations among this population [34]. Similarly, when addressing the Hispanic/Latinx subgroup, it was important for the item to include most, if not all, of the Hispanic and Latinx origins, as each group differs in many ways [35,36]. One question assessed if the person was mandated to take the COVID-19 vaccine, and two additional questions assessed the person’s trust in a medical professional for COVID-19 vaccine information and encouragement. Thirty items assessed the constructs of MTM, of which fifteen items assessed the initiation construct and fifteen items assessed the sustenance construct.

### 2.4. Survey Translation

The survey was written in English and translated into Spanish to ensure there was access to the predominant languages of the Hispanic and Latinx populations. The survey was then retranslated back to English to ensure proper translation of survey content.

### 2.5. Face and Content Validity

The instrument was validated by six experts in public health and the Hispanic and Latinx populations to ensure content validity. The experts included professors with doctorate degrees in public health and/or the MTM theoretical framework, community partners that focused on and worked with the Hispanic and Latinx populations, and individuals who were knowledgeable about COVID-19 vaccination based on their involvement with vaccine distribution. After validation by experts, the instrument had a Flesch Reading Ease score of 52.3 and a Flesch–Kincaid Grade Level of 9.9 overall. The instrument was thoroughly reviewed by experts and community members to ensure that face and content validity were being measured appropriately.

### 2.6. Data Collection

The survey instrument was administered via three routes: (1) a web-based survey tool via Qualtrics, (2) in-person outreach via paper surveys and flyers with QR codes to the web-based survey tool, and (3) calls via Qualtrics Sample Services. To administer the survey via the web-based survey tool, participants were recruited through community contacts that had an established connection with the Hispanic and Latinx populations to ensure participants had trust and confidence in the individuals recruiting for and/or administering the survey. A recruitment email and flyer were provided in both English and Spanish. Participants were also recruited at in-person events with a local nonprofit organization. At in-person events, such as pop-up vaccination clinics, education sessions, and outreach events throughout Nevada, the recruitment flyer was displayed for participation, and paper surveys were available. The researcher and/or other volunteers recruited participants by distributing paper surveys and flyers. For the completed paper surveys, the researcher inputted all answers reported on paper directly onto the Qualtrics survey. The researcher also employed Qualtrics Sample Services to perform the data collection to reach the ideal sample size. The Qualtrics Sample Services delivery team managed the data collection process and invited respondents that met the geographic and demographic restrictions to complete the online survey.

### 2.7. Construct Validity

Confirmatory factor analysis was used to assess the construct validity by using the maximum likelihood estimation of all MTM subscales being studied, including advantages, disadvantages, behavioral confidence, changes in the physical environment, emotional transformation, practice for change, and changes in the social environment. This was determined if each construct yielded a single-factor solution, factor loading values greater than 0.384, and an Eigenvalue that was greater than or equal to 1 [37].

### 2.8. Reliability

Cronbach’s alphas were used to determine the internal consistency reliability for each MTM construct. These values were compared to a value of 0.70 or higher to be considered acceptable [38,39].

### 2.9. Data Analysis

The survey data from Qualtrics were further analyzed in SPSS (Version 27.0, IBM, Armonk, NY, USA). Descriptive statistical analysis was conducted for all study variables. Counts and frequencies were reported for all demographic characteristics and categorical study variables. Continuous study variables reported means and standard deviations. The demographic characteristics of age, race, gender, education level, religion, income, and employment status served as covariates in the multivariate data analysis plan.

A zero-order correlation matrix was conducted among the construct variables to identify if there were any significant, simple bivariate relationships between the theoretical constructs and both the initiation and sustenance for the hesitant and non-hesitant groups.

Hierarchical multiple regression was used to “control” for certain variables among different groups to see if adding variables improved the model’s capacity to predict the likelihood of getting the COVID-19 vaccine and/or the second dose/booster dose [40]; this was used to study the hesitant and non-hesitant groups and their relationship with the two outcome variables of initiation and sustenance, which formed four models. The significance level was set at 0.05 for all data analyses, and 95% confidence intervals were reported as applicable.

### 2.10. Ethical Approval

This study was submitted for approval to the University of Nevada, Las Vegas Institutional Review Board (IRB). The study was first approved as exempt on 3 May 2022 (UNLV-2022-192). It was then approved for its first modification to the protocol, informed consent form, and recruitment materials on 17 June 2022. The final modification was approved on 20 July 2022, for an addition to the recruitment and data collection strategy. Participants were required to provide consent to participation in the survey by clicking on the next button in the electronic version and by continuing the survey in the paper version. Participants were allowed to choose to withdraw from the survey at any time. For Spanish-speaking participants, the consent form and survey were presented “in [a] language understandable to the subject” [35,41]. All procedures to conduct the research involving human subjects followed the IRB ethical standards.

## 3. Results

### 3.1. Confirmatory Factor Analysis for Construct Validity

Confirmatory factor analysis was used to assess the construct validity by using the maximum likelihood estimation of all MTM subscales being studied, including advantages, disadvantages, behavioral confidence, changes in the physical environment, emotional transformation, practice for change, and changes in the social environment. Confirmatory factor analysis revealed that each MTM subscale generated a single-factor solution, with most having factor loadings greater than 0.326 and an Eigenvalue greater than or equal to 1 [37]. All but one item met the critical value of 0.326 for factor loadings [37]. Of those that met the critical value, the minimum factor loading was 0.615 and the maximum factor loading was 0.999. The majority of factor loadings were over double the critical value, indicating that these were high factor loadings. The item that did not meet the critical value was the question “Do you believe the COVID-19 vaccine is accessible for you to get it if you wanted it?” under the behavioral confidence construct, with a factor loading of 0.308.

### 3.2. Descriptive Statistics of Demographic Variables

The final sample size included 231 participants. Results from the descriptive statistical analysis are displayed in Table 1. The mean age of participants was 37.83 ± 14.14 years. The majority of participants identified as female (*n* = 160, 69.3%). Because all participants identified as being of Hispanic or Latinx descent, the Hispanic/Latinx identity that was most associated with participants was Mexican (*n* = 146, 63.2%). The highest level of education achieved by most participants was “some college” (*n* = 94, 40.7%) and high school (*n* = 75, 32.5%). Of all religions presented, approximately a third of participants identified as believing in Catholicism (*n* = 79, 34.2%); unaffiliated with any religion (*n* = 69, 29.9%) had the second highest number of participants. More than half of the participants were employed (*n* = 138, 59.7%), where the highest reported individual incomes were USD 25,000 to USD 49,999 (*n* = 85, 36.8%) and USD 50,000 to USD 74,999 (*n* = 53, 22.9%). The mean average number of people living in one household was 3.22 ± 1.57 people. In addition, most participants reported their marital status as single (*n* = 84, 36.4%) or married (*n* = 74, 32.0%). Most participants reported possessing health insurance (*n* = 182, 78.8%). Of the participants who had responded to the optional questions, participants reported their political affiliation as either Republican (*n* = 46, 19.9%), Democratic (*n* = 82, 35.5%), Independent (*n* = 59, 25.5%), other (*n* = 17, 7.4%), or prefer not to answer (*n* = 21, 9.1%). The second optional question asked about current citizenship status, in which the vast majority of respondents reported being a citizen of the United States (*n* = 206, 89.2%).

Most importantly for further data analysis, 36.4% of participants expressed hesitancy to take the COVID-19 vaccine (*n* = 84) and 63.6% of participants did not express hesitancy to take the COVID-19 vaccine (*n* = 147). A little over half of the participants had received at least one dose of the COVID-19 vaccine (*n* = 136, 58.9%), which slightly decreased the number of participants who had completed the series of the COVID-19 vaccine (*n* = 127, 55.0%), meaning they received at least two doses of the Pfizer or Moderna vaccine or one dose of the Janssen vaccine. While 69.7% of participants reported having a trusted medical provider to provide COVID-19 vaccine information (*n* = 161), more than half of participants reported not having been encouraged by their medical provider to take the COVID-19 vaccine (*n* = 133, 57.6%).

### 3.3. Descriptive Statistics of Construct Variables

Table 2 displays the descriptive statistics of the MTM constructs as the independent variables and the dependent variables of initiation and sustenance. Their significance was assessed among the participants who expressed hesitancy and did not express hesitancy toward taking the COVID-19 vaccine. Mean scores are reported in Table 2. When comparing mean scores of all variables between the vaccine-hesitant and non-vaccine-hesitant groups, mean values for all constructs measured significantly higher among the non-hesitant group for each variable, except for the participatory dialogue: disadvantages. Only with the participatory dialogue: disadvantages construct variable did results indicate a mean score that was higher among vaccine-hesitant individuals compared to non-vaccine individuals, indicating vaccine-hesitant individuals agree with more of the disadvantages of the COVID-19 vaccine over the advantages.

Cronbach’s alpha was reported for all independent variables, or the MTM constructs, among all participants to determine internal consistency reliability for each MTM construct. These values are reported in Table 2. Cronbach’s alpha values that were 0.70 or higher were considered acceptable [38,39]. All Cronbach’s alpha values for each MTM construct variable were above 0.70, where values ranged from the lowest value of 0.773 for behavioral confidence to the highest value of 0.992 for emotional transformation. Because all Cronbach’s alpha values were above 0.70, these values were deemed acceptable. Behavioral confidence had the lowest Cronbach’s alpha value of 0.773, which is still deemed acceptable, but is a lower value compared to the other MTM constructs.

### 3.4. Zero-Order Correlation Matrix of Construct Variables

The results of the zero-order correlation matrix to describe the bivariate associations between the MTM construct variables among vaccine-hesitant and non-vaccine-hesitant individuals are described in Table 3 for initiation and in Table 4 for sustenance. Based on Table 3 results, initiation was only statistically related to participatory dialogue: advantages−disadvantages (r = 0.691, *p* < 0.001) and behavioral confidence (r = 0.636, *p* < 0.001) for vaccine-hesitant individuals. The magnitude of associations between initiation, participatory dialogue, and behavioral confidence constructs were nearly similar. Among non-vaccine-hesitant individuals, initiation was statistically related to participatory dialogue: advantages−disadvantages (r = 0.606, *p* < 0.001), behavioral confidence (r = 0.762, *p* < 0.001), and changes in the physical environment (r = 0.587, *p* < 0.001). Initiation and behavioral confidence had the highest magnitude of association compared to the other MTM relationships among non-vaccine-hesitant individuals.

Based on Table 4 results, sustenance was statistically related to emotional transformation (r = 0.530, *p* < 0.001), practice for change (r = 0.382, *p* < 0.001), and changes in the social environment (r = 0.248, *p* = 0.025) for vaccine-hesitant individuals. Similarly, among non-vaccine-hesitant individuals, sustenance was statistically related to emotional transformation (r = 0.816, *p* < 0.001), practice for change (r = 0.632, *p* < 0.001), and changes in the social environment (r = 0.658, *p* < 0.001). Among both vaccine-hesitant and non-vaccine-hesitant individuals, sustenance and emotional transformation had the highest magnitude of association compared to the other MTM relationships.

### 3.5. Hierarchical Multiple Regression among Construct Variables and Covariates

The hierarchical multiple regression modeling results among both groups are displayed in Table 5 for the initiation of the COVID-19 vaccine and Table 6 for the sustenance of the COVID-19 vaccine. Individual characteristics of age, race, gender, education level, religion, income, and employment status were also included as covariates in the models due to their historical identification of having an effect on COVID-19 vaccination uptake.

Among vaccine-hesitant individuals, participatory dialogue and behavioral confidence explained 63.0% of the variability in the initiation of COVID-19 vaccine acceptance behavior (adjusted R^2^ = 0.630, F(9,73) = 16.520, *p* < 0.001) (Table 5). After controlling for covariates, participatory dialogue (b = 0.113, *p* < 0.001) and behavioral confidence (b = 0.358, *p* < 0.001) displayed statistically significant associations with the initiation of COVID-19 vaccine acceptance. Additionally, one individual characteristic of income, specifically an income range of USD 25,000 to USD 49,999, displayed significant results as a predictor of initiation. This income range is associated with a 0.486 increase in initiation score (b = 0.486, *p* = 0.007) among vaccine-hesitant individuals when compared to other income ranges lower than USD 25,000 and higher than USD 49,999.

Similar to vaccine-hesitant individuals, among non-vaccine-hesitant individuals, a hierarchical multiple regression model including all covariates, participatory dialogue, and behavioral confidence explained 63.2% of the variability in the initiation of COVID-19 vaccine acceptance behavior (adjusted R^2^ = 0.632, F(9,132) = 27.959, *p* < 0.001) (Table 5). After controlling for covariates, similar to the model of vaccine-hesitant individuals, participatory dialogue (b = 0.072, *p* < 0.001) and behavioral confidence (b = 0.206, *p* < 0.001) displayed significant associations with the initiation of COVID-19 vaccine acceptance. Another individual characteristic of age displayed statistically significant results as a predictor of initiation, whereas age was associated with a 0.017 increase in initiation score (b = 0.017, *p* = 0.003) among non-vaccine-hesitant individuals.

In the examination of the sustenance component, a hierarchical multiple regression model including all covariates and emotional transformation explained 37.4% of the variability in the sustenance of COVID-19 vaccine acceptance behavior (adjusted R^2^ = 0.374, F(8,73) = 7.045, *p* < 0.001) (Table 6). After controlling for covariates, emotional transformation (b = 0.087, *p* < 0.001) displayed a statistically significant association with the sustenance of COVID-19 vaccine acceptance. Only the individual characteristic of age among the vaccine-hesitant individuals displayed significant results as a predictor of sustenance, whereas age was associated with a 0.019 decrease in sustenance score (b = −0.019, *p* = 0.004).

Similar to vaccine-hesitant individuals, among non-vaccine-hesitant individuals, a hierarchical multiple regression model including all covariates and emotional transformation explained 66.4% of the variability in the sustenance of COVID-19 vaccine acceptance behavior (adjusted R^2^ = 0.664, F(8,133) = 35.801, *p* < 0.001) (Table 6). After controlling for covariates, emotional transformation (b = 0.177, *p* < 0.001) displayed a statistically significant association with the sustenance of COVID-19 vaccine acceptance. No other individual characteristic showed significant associations for sustenance among non-vaccine-hesitant individuals.

## 4. Discussion

### 4.1. Interpretation of Findings

The COVID-19 vaccine was identified by participants as an effective public health tool to slow the spread of disease throughout the community. However, of the 231 respondents, 36.4% (*n* = 84) of individuals expressed hesitancy to take the COVID-19 vaccine. This finding was similar to that of various studies that found approximately a third of the Hispanic population is very hesitant to get vaccinated [3,18]. A response rate of 36.4% of our sample population expressing vaccine hesitancy indicates that there is a strong need for public health professionals to encourage COVID-19 vaccine uptake among vaccine-hesitant Hispanics and Latinxs to ensure we are able to reach a herd immunity threshold that will slow the spread of disease and put an end to the pandemic.

Our study results provide further support that two of the three MTM initiation constructs, specifically participatory dialogue and behavioral confidence, were shown to be significant in explaining the intent of initiating the COVID-19 vaccine for both vaccine-hesitant and non-vaccine-hesitant individuals. As presented by the results in Table 2, the mean score for participatory dialogue among vaccine-hesitant individuals was −6.071 ± 4.834. On the contrary, the mean score for participatory dialogue among non-vaccine-hesitant individuals was +2.421 ± 4.785, indicating that these participants believed more in the advantages of the COVID-19 vaccine and less in the disadvantages. These lower mean scores are also supported by previous survey results presented by Wanin that only 34% of Latinx participants trusted the COVID-19 vaccine’s safety and nearly 40% trusted the COVID-19 vaccine’s effectiveness [42]. With the introduction of a novel COVID-19 vaccine, these mean scores show that there are still some hesitancies about the advantages among both vaccine-hesitant and non-vaccine-hesitant individuals; however, these mean scores highlight that there are more hesitancies among the vaccine-hesitant individuals. They further highlight a need to focus on the advantages of the COVID-19 vaccine when addressing this particular construct among the Hispanic and Latinx populations. Since the COVID-19 vaccine was available free to all, the third MTM construct of changes in the physical environment may not have played a significant role in our study. Perhaps in the future, when COVID-19 boosters are not available for free, this construct may play a greater role.

Among both the vaccine-hesitant and non-vaccine-hesitant groups, behavioral confidence was highlighted as an important construct in predicting the initiation of COVID-19 vaccine acceptance. According to Reverby, there is a lack of confidence and trust in vaccine availability, side effects, and studies performed on the COVID-19 vaccine because there are myths and misconceptions that the vaccine is used to harm or track people, which can cause more fear than confidence [43]. Behavioral confidence then highlights the need to build trust within the Hispanic and Latinx communities when encouraging vaccine acceptance behaviors. Ensuring Hispanic and Latinx populations receive more proper education and information from credible sources to build confidence in receiving the vaccine will help to increase vaccination uptake.

Only one of the three MTM sustenance constructs, specifically emotional transformation, was shown to be significant in explaining the intent of sustaining the COVID-19 vaccine for both vaccine-hesitant and non-vaccine-hesitant individuals. Similar to the study by Salgado de Snyder et al., the construct of emotional transformation could easily affect the sustenance of receiving COVID-19 vaccines and/or a routine vaccine due to fear or lack of ability to overcome these challenges [44]. Among the Mexican men who were surveyed, a fear of needles or side effects, being lazy and irresponsible, not caring or needing to get vaccinated, and inconvenience or a lack of time to get vaccinated due to conflicting work schedules were described [44]. Additionally, Hamel et al. and Dawson et al. also highlighted challenges to not receiving a second dose of the COVID-19 vaccine that also included the cost of the vaccine and immigration status [19,45]. This further supports a need to address solutions to overcoming these challenges to getting vaccinated, which may include setting up vaccination clinics at various locations convenient to the individual or advocating for policy changes that will allow for employees to take paid time out of their work schedule to get vaccinated and recover if side effects do take a toll on their ability to continue working. Right now, long-term behavior change regarding the COVID-19 vaccine is not apparent. While the two constructs of sustenance, namely practice for change and change in the social environment, were not found to be significant in the present study, in the future, more regular boosters may be required, thus necessitating the importance of these two sustenance constructs. The time period in which this study was conducted was rather limited, and the other two constructs of MTM (practice for change and changes in the social environment) may play a greater role if regular boosters are necessary for protection against COVID-19. Nonetheless, these findings can be used for future research when planning MTM-based implementation strategies to increase COVID-19 vaccine acceptance behavior specifically for Hispanic and Latinx populations.

Similar to various studies, the covariate of age was shown as a significant predictor of COVID-19 vaccine acceptance behavior, particularly for the initiation of the vaccine among non-vaccine-hesitant individuals and the sustenance of the vaccine among vaccine-hesitant individuals. This finding is further supported by results from the December 2020 Kaiser Family Foundation COVID-19 Vaccine Monitor that found Hispanic adults that were older than 50 years had more trust in the vaccine and were more likely to take the vaccine compared to their younger counterparts, who reported more vaccine hesitancy and lack of trust in government officials [18]. Younger adults may not continue with follow-up of the second dose or booster dose. It may be that younger age groups believe they are healthy and do not need the vaccine.

Income was also shown as a significant predictor of initiation of the COVID-19 vaccine among vaccine-hesitant participants. This displayed that as one gets employed and income increases, there is more of an increase in individuals initiating the COVID-19 vaccine series. One explanation for this is that working individuals do not want to get sick and be forced to take the day off, losing out on pay. Getting the COVID-19 vaccine lowers one’s chance of getting seriously ill and hospitalized from COVID-19, allowing one to keep working to make their income.

### 4.2. Implications for Practice

Based on study results, it is evident there is a need for theory-based interventions and messaging to address vaccine hesitancy and barriers that affect COVID-19 vaccine acceptance among the Hispanic and Latinx populations. As described by Salmon et al., trusted voices within their communities provide a heavy influence on decision-making among Hispanic and Latinx communities [23]. Therefore, hosting group interventions that are led by trusted community members and/or leaders in public health in trusted locations such as a school or community center will encourage participation in the study. It may also be beneficial to employ non-U.S. citizens to conduct research or lead intervention strategies to gain trust among the non-U.S. citizen communities. Many of the Hispanic and Latinx communities have had negative historical experiences with racism and medical exclusions, therefore emphasizing the need for a trusted resource to lead the intervention.

To influence the MTM constructs of participatory dialogue, behavioral confidence, and emotional transformation, small group and one-on-one discussions may be beneficial to addressing concerns, providing demonstrations for researching credible sources of knowledge, and motivating participants to overcome challenges to getting vaccinated. These interventions should also be available in the Spanish language, whether it be a Spanish speaker or with Spanish-translated resources to ensure that communication is continuous and in the participant’s native language. Additionally, using a multimodal approach, such as using technology and social media, would help to continue the discussions started in the intervention and to address additional concerns participants may have.

Based on our results, the use of a theory-based intervention is critical to ensure the study uses a structured model that has been extensively studied and proven to be predictive of the health behavior we are trying to change. By using a theory-based approach, we have proof that these constructs are predictive of a health behavior change.

### 4.3. Strengths of the Study

To our knowledge, this is the first study that utilized a theory-based survey instrument to assess COVID-19 vaccine acceptance behavior among the Hispanic and Latinx populations. This study design was also very beneficial in providing relatively quick results, was particularly low cost, and provided the ability to easily evaluate this particular population in a short amount of time. The survey was written in English, then translated into Spanish and retranslated back to English to ensure the translation was an accurate reflection of the same verbiage of questions.

### 4.4. Limitations

This study had some limitations. The study utilized a cross-sectional study which may not determine if an association equals causation or the directionality of the outcome. Additionally, as with any self-reported survey study design, one limitation was response bias. Recruitment bias may have occurred due to the difficulty of obtaining participants early in the recruitment stages. Another limitation was that the survey instrument had a Flesch Reading Ease score of 52.3 and a Flesch–Kincaid Grade Level of 9.9, which made the survey fairly difficult to read. One recommendation for future research would be to change the survey instrument by editing the survey for a lower readability score and lower grade level score to ensure more people are able to understand and take the survey, especially if participants are not native English speakers. Upon editing, the survey instrument can also be further edited to assess the prediction of other routine immunizations, such as influenza; measles, mumps, and rubella (MMR); and human papillomavirus (HPV). The sample collected contained responses from predominantly females (69%) and people of Mexican identity (63.2%), all of whom resided in Nevada. This limits the generalizability of the study findings to all genders and other Hispanic/Latinx identities outside of Nevada. Another quantitative study design that would help to generalize the study’s findings could be conducted using a larger population sample; however, a qualitative study design utilizing interviews and focus groups may help to gain a deeper understanding of the participatory dialogue and behavioral confidence that would affect the initiation of the vaccine, as well as the emotional transformation of the sustenance of the vaccine. Despite the limitations, this study provided a foundation for theory-based research among the Hispanic and Latinx communities to understand what factors predict COVID-19 vaccine acceptance behaviors, and it can be tailored in future research and interventions.

## 5. Conclusions

The COVID-19 pandemic has had a significantly disproportionate negative impact on the Hispanic and Latinx populations. Vaccine hesitancy and access to vaccines have prevented the rapid uptake of the COVID-19 vaccine. One of the MTM constructs, emotional transformation for sustenance, must be influenced by solutions to overcoming the challenges in getting vaccinated, as this plays a large factor in why people who may be accepting of the vaccine ultimately choose not to receive the vaccine. Addressing solutions that include advocating for policy change may be the most effective, including policy changes that will allow for employees to take paid time out of their work schedule to get vaccinated and recover if side effects take a toll on their ability to continue working. This may be particularly challenging, especially for ethnic minorities who may feel like their voices are not heard; however, these policies may help to provide more equitable access to vaccines and overall increase trust in policy makers.

This study aimed to assess the MTM’s ability to predict COVID-19 vaccine acceptance behavior among the Hispanic and Latinx populations in Nevada. Results from this study provide evidence that the MTM is a useful tool in predicting COVID-19 vaccine acceptance behavior among Hispanics and Latinxs in Nevada and can be used to influence vaccine uptake behaviors. Interventions and messaging to encourage vaccine uptake are crucial to address COVID-19 vaccine hesitancy to promote its rapid uptake, and the use of MTM can be effective in this development to ensure Hispanics and Latinxs are protected against the spread of COVID-19.

## Figures and Tables

**Table 1:** Descriptive characteristics of the study sample (*n* = 231).

Characteristic	M (SD)	*n* (%)
Age (in years)	37.83 (14.141)	
Gender		
Male		69 (29.9)
Female		160 (69.3)
Other		0 (0.0)
Hispanic/Latinx Identity		
Argentinian		5 (2.2)
Bolivian		0 (0.0)
Chilean		2 (0.9)
Colombian		4 (1.7)
Costa Rican		2 (0.9)
Cuban		7 (3.0)
Dominican		2 (0.9)
Ecuadorian		1 (0.4)
Guatemalan		4 (1.7)
Honduran		4 (1.7)
Mexican		146 (63.2)
Nicaraguan		2 (0.9)
Panamanian		1 (0.4)
Paraguayan		0 (0.0)
Peruvian		1 (0.4)
Puerto Rican		18 (7.8)
Salvadoran		4 (1.7)
Uruguayan		0 (0.0)
Venezuelan		0 (0.0)
Other Central American		0 (0.0)
Other South American		3 (1.3)
All other Hispanic or Latino		19 (8.2)
Prefer not to answer		4 (1.7)
Highest level of education		
Less than high school		5 (2.2)
High school		75 (32.5)
Some college		94 (40.7)
Bachelor’s degree or higher		53 (22.9)
Religion		
Buddhism		2 (0.9)
Catholicism		79 (34.2)
Judaism		4 (1.7)
Mormonism		3 (1.3)
Orthodox Christian		7 (3.0)
Other Christianity		40 (16.9)
Protestant		8 (3.5)
Unaffiliated with any religion		69 (29.9)
Other		17 (7.4)
Annual individual income		
USD 0 to USD 9999		15 (6.5)
USD 10,000 to USD 24,999		33 (14.3)
USD 25,000 to USD 49,999		85 (36.8)
USD 50,000 to USD 74,999		53 (22.9)
USD 75,000 to USD 99,999		28 (12.1)
USD 100,000 to USD 149,999		14 (6.1)
Over USD 150,000		1 (0.4)
Current employment status		
Employed		138 (59.7)
Self-employed		26 (11.3)
Laid-off/Furloughed		0 (0.0)
Retired		12 (5.2)
Homemaker		20 (8.7)
Unreported employment		2 (0.9)
Unemployed		27 (11.7)
Other		4 (1.7)
Number of people living in household	3.22 (1.567)	
Marital status		
Single		84 (36.4)
Married		74 (32.0)
Divorced		31 (13.4)
Widowed		1 (0.4)
Separate		5 (2.2)
Never married		6 (2.6)
In a civil union or registered domestic partnership		11 (4.8)
A member of an unmarried couple		17 (7.4)
Possesses health insurance		
Yes		182 (78.8)
No		47 (20.3)
Political affiliation (optional to answer)		
Republican		46 (19.9)
Democratic		82 (35.5)
Independent		59 (25.5)
Other		17 (7.4)
Prefer not to answer		21 (9.1)
Current citizenship status (optional to answer)		
Is a citizen of the United States		206 (89.2)
Not a citizen of the United States		12 (5.2)
Prefer not to answer		6 (2.6)
Expresses hesitancy to taking COVID-19 vaccine		
Yes		84 (36.4)
No		147 (63.6)
Received at least one dose of the COVID-19 vaccine		
Yes		136 (58.9)
No		95 (41.1)
Completed series of COVID-19 vaccine		
Yes		127 (55.0)
No		104 (45.0)
Has a trusted provider provided COVID-19 vaccine information infmation		
Yes		161 (69.7)
No		68 (29.4)
Has been encouraged by a medical provider to take the COVID-19 vaccine		
Yes		96 (41.6)
No		133 (57.6)

**Table 2:** Descriptive characteristics of study variables (*n* = 231).

Variable	Vaccine-Hesitant Individuals (*n* = 84)			Vaccine Non-Hesitant Individuals (*n* = 147)			All Participants (*n* = 231)	
	Possible Range	Observed Range	Mean (SD)	Possible Range	Observed Range	Mean (SD)	Cronbach’s Alpha	*p*-Value
Initiation	0–4	0–4	0.843 (1.1841)	0–4	0–4	3.056 (1.378)		<0.001
Participatory dialogue: advantages	0–12	0–9	3.083 (2.617)	0–12	0–12	7.545 (3.440)	0.960	<0.001
Participatory dialogue: disadvantages	0–12	2–12	9.155 (2.659)	0–12	0–12	5.124 (2.850)	0.841	0.002
Participatory dialogue: advantages–disadvantages	−12–+12	−12–+7	−6.071 (4.834)	−12–+12	−12–+12	2.421 (4.785)		<0.001
Behavioral confidence	0–12	0–9	4.361 (1.664)	0–12	0–12	8.570 (3.351)	0.773	<0.001
Changes in the physical environment	0–20	0–20	12.928 (5.055)	0–20	0–20	14.278 (5.117)	0.870	<0.001
Sustenance	0–4	0–4	0.634 (0.988)	0–4	0–4	2.722 (1.465)		<0.001
Emotional transformation	0–24	0–23	7.277 (5.315)	0–24	0–24	16.133 (7.0653)	0.992	<0.001
Practice for change	0–20	0–20	7.634 (6.093)	0–20	0–20	13.090 (5.390)	0.901	<0.001
Changes in the social environment	0–12	0–12	5.061 (3.923)	0–12	0–12	8.069 (3.363)	0.907	<0.001

Estimates attained for significance testing are based on independent *t*-tests.

**Table 3:** Zero-order correlation matrix of study variables for initiation of COVID-19 vaccination behavior.

**Vaccine-Hesitant Individuals (*n* = 84)**				
**Construct**	**Initiation**	**Participatory Dialogue**	**Behavioral Confidence**	**Changes in the Physical Environment**
1. Initiation	–	0.691 ** (*p* < 0.001)	0.636 ** (*p* < 0.001)	−0.165 (*p* = 0.136)
2. Participatory dialogue: advantages–disadvantages		–	0.411 ** (*p* < 0.001)	−0.320 ** (*p* = 0.003)
3. Behavioral confidence			–	0.202 (*p* = 0.067)
4. Changes in the physical environment				–
**Vaccine Non-Hesitant Individuals (*n* = 147)**				
**Construct**	**Initiation**	**Participatory Dialogue**	**Behavioral Confidence**	**Changes in the Physical Environment**
1. Initiation	–	0.606 ** (*p* < 0.001)	0.762 ** (*p* < 0.001)	0.587 ** (*p* < 0.001)
2. Participatory dialogue advantages–disadvantages		–	0.568 ** (*p* < 0.001)	0.361 ** (*p* < 0.001)
3. Behavioral confidence			–	0.696 ** (*p* < 0.001)
4. Changes in the physical environment				–

** Correlation is significant at the 0.01 level (2-tailed).

**Table 4:** Zero-order correlation matrix of study variables for the sustenance of COVID-19 vaccination behavior.

**Vaccine-Hesitant Individuals (*n* = 84)**				
**Construct**	**Sustenance**	**Emotional Transformation**	**Practice for Change**	**Changes in the Social Environment**
1. Sustenance	–	0.530 ** (*p* < 0.001)	0.382 ** (*p* < 0.001)	0.248 * (*p* = 0.025)
2. Emotional transformation		–	0.541 ** (*p* < 0.001)	0.327 ** (*p* = 0.003)
3. Practice for change			–	0.687 ** (*p* < 0.001)
4. Changes in the social environment				–
**Vaccine Non-Hesitant Individuals (*n* = 147)**				
**Construct**	**Sustenance**	**Emotional Transformation**	**Practice for Change**	**Changes in the Social Environment**
1. Sustenance	–	0.816 ** (*p* < 0.001)	0.632 ** (*p* < 0.001)	0.658 ** (*p* < 0.001)
2. Emotional transformation		–	0.789 ** (*p* < 0.001)	0.807 ** (*p* < 0.001)
3. Practice for change			–	0.859 ** (*p* < 0.001)
4. Changes in the social environment				–

** Correlation is significant at the 0.01 level (2-tailed). * Correlation is significant at the 0.05 level (2-tailed).

**Table 5:** Multiple regression models for initiation of COVID-19 vaccination among hesitant and non-hesitant participants.

**Hesitant Participants**	***b***	**S.E.**	**β**	***p***	**LBCI**	**UBCI**
Age	0.003	0.006	0.035	0.623	−0.009	0.015
Mexican (reference: non-Mexican)	−0.040	0.164	−0.017	0.807	−0.367	0.287
Female (reference: male)	0.129	0.184	0.051	0.485	−0.238	0.496
Some college (reference: high school education or less, or bachelor’s degree and higher)	−0.042	0.172	−0.018	0.809	−0.385	0.302
Catholicism (reference: non-Catholicism)	−0.004	0.192	−0.001	0.985	−0.387	0.379
USD 25,000 to USD 49,999 (reference: lower and higher income than USD 25,000 to USD 49,999)	0.486	0.175	0.193	0.007	0.136	0.835
Employed (reference: other employment or non-employed)	0.099	0.165	0.042	0.550	−0.230	0.428
Participatory dialogue advantages–disadvantages	0.113	0.021	0.461	<0.001	0.071	0.155
Behavioral confidence	0.358	0.059	0.503	<0.001	0.241	0.475
Changes in the physical environment	−0.032	0.019	−0.135	0.099	−0.069	0.006
Model statistics including predictors of covariates, participatory dialogue, and behavioral confidence: R^2^ = 0.671, adjusted R^2^ = 0.630, F_(9,73)_ = 16.520, *p* < 0.001						
**Non-Hesitant Participants**	***b***	**S.E.**	**Β**	***p***	**LBCI**	**UBCI**
Age	0.017	0.006	0.172	0.003	0.006	0.028
Mexican (reference: non-Mexican)	−0.003	0.159	−0.001	0.983	−0.318	0.311
Female (reference: male)	0.093	0.159	0.031	0.557	−0.220	0.407
Some college (reference: high school education or less, or bachelor’s degree and higher)	−0.017	0.159	−0.006	0.915	−0.330	0.297
Catholicism (reference: non-Catholicism)	−0.057	0.152	−0.020	0.707	−0.357	0.243
USD 25,000 to USD 49,999 (reference: lower and higher income than USD 25,000 to USD 49,999)	0.124	0.149	0.044	0.408	−0.171	0.419
Employed (reference: other employment or non-employed)	0.175	0.156	0.062	0.263	−0.133	0.483
Participatory dialogue advantages–disadvantages	0.072	0.018	0.249	<0.001	0.035	0.108
Behavioral confidence	0.206	0.034	0.502	<0.001	0.139	0.274
Changes in the physical environment	0.031	0.019	0.116	0.109	−0.007	0.069
Model statistics including predictors of covariates, participatory dialogue, and behavioral confidence: R^2^ = 0.656, adjusted R^2^ = 0.632, F_(9,132)_ = 27.959, *p* < 0.001						

S.E. = standard error of the estimate; LBCI = lower bound of the 95% confidence interval; UBCI = upper bound of the 95% confidence interval.

**Table 6:** Multiple regression models for the sustenance of COVID-19 vaccination among hesitant and non-hesitant participants.

**Hesitant Participants**	***b***	**S.E.**	**β**	***p***	**LBCI**	**UBCI**
Age	−0.019	0.006	−0.275	0.004	−0.032	−0.006
Mexican (reference: non-Mexican)	0.129	0.185	0.066	0.487	−0.239	0.497
Female (reference: male)	−0.212	0.209	−0.100	0.314	−0.628	0.205
Some college (reference: high school education or less, or bachelor’s degree and higher)	−0.269	0.181	−0.137	0.140	−0.630	0.091
Catholicism (reference: non-Catholicism)	0.097	0.211	0.045	0.646	−0.323	0.518
USD 25,000 to USD 49,999 (reference: lower and higher income than USD 25,000 to USD 49,999)	−0.115	0.194	−0.055	0.554	−0.501	0.271
Employed (reference: other employment or non-employed)	0.079	0.189	0.040	0.679	−0.299	0.456
Emotional transformation	0.087	0.020	0.470	<0.001	0.046	0.127
Practice for change	0.018	0.023	0.114	0.416	−0.027	0.063
Changes in the social environment	−0.004	0.032	−0.017	0.890	−0.067	0.058
Model statistics including predictors of covariates and emotional transformation: R^2^ = 0.436, adjusted R^2^ = 0.374, F_(8,73)_ = 7.045, *p* < 0.001						
**Non-Hesitant Participants**	***b***	**S.E.**	**β**	***p***	**LBCI**	**UBCI**
Age	0.006	0.006	0.061	0.294	−0.006	0.018
Mexican (reference: non-Mexican)	−0.157	0.164	−0.048	0.341	−0.481	0.167
Female (reference: male)	−0.111	0.162	−0.035	0.492	−0.432	0.209
Some college (reference: high school education or less, or bachelor’s degree and higher)	0.089	0.167	0.030	0.594	−0.241	0.419
Catholicism (reference: non-Catholicism)	0.146	0.157	0.048	0.356	−0.166	0.457
USD 25,000 to USD 49,999 (reference: lower and higher income than USD 25,000 to USD 49,999)	0.212	0.153	0.071	0.166	−0.090	0.515
Employed (reference: other employment or non-employed)	0.129	0.160	0.043	0.421	−0.187	0.446
Emotional transformation	0.177	0.019	0.850	<0.001	0.139	0.215
Practice for change	−0.015	0.028	−0.054	0.606	−0.070	0.041
Changes in the social environment	0.000	0.048	−0.001	0.994	−0.096	0.095
Model statistics including predictors of covariates and emotional transformation: R^2^ = 0.683, adjusted R^2^ = 0.664, F_(8,133)_ = 35.801, *p* =< 0.001						

S.E. = standard error of the estimate; LBCI = lower bound of the 95% confidence interval; UBCI = upper bound of the 95% confidence interval.

## Data Availability

Data are available on request with the permission of T.N.
